# Design of the Endobronchial Valve for Emphysema Palliation Trial (VENT): a non-surgical method of lung volume reduction

**DOI:** 10.1186/1471-2466-7-10

**Published:** 2007-07-03

**Authors:** Charlie Strange, Felix JF Herth, Kevin L Kovitz, Geoffrey McLennan, Armin Ernst, Jonathan Goldin, Marc Noppen, Gerard J Criner, Frank C Sciurba, the VENT Study Group

**Affiliations:** 1 Division of Pulmonary and Critical Care Medicine, Medical University of South Carolina, Charleston, SC, USA; 2 Department of Pneumology and Critical Care Medicine, Thoraxklinik am Universtaetsklinikum, Heidelberg, Germany; 3 Section of Pulmonary, Critical Care and Environmental Medicine, Tulane University Health Sciences Center, New Orleans, LA, USA; 4 Colleges of Medicine and Engineering, University of Iowa, Iowa City, Iowa, USA; 5 Division of Thoracic Surgery and Interventional Pulmonary, Beth Israel Deaconess Medical Center, Harvard Medical School, Boston, MA, USA; 6 Department of Radiology, David Geffen School of Medicine at UCLA, Los Angeles, CA, USA; 7 Interventional Endoscopy Clinic, University Hospital AZ-VUB, Brussels, Belgium; 8 Division of Pulmonary and Critical Care Medicine, Temple University School of Medicine, Philadelphia, PA, USA; 9 Division of Pulmonary and Critical Care Medicine, Department of Medicine, University of Pittsburgh School of Medicine, USA; 10 the VENT Study Group is provided at the end of the article

## Abstract

**Background:**

Lung volume reduction surgery is effective at improving lung function, quality of life, and mortality in carefully selected individuals with advanced emphysema. Recently, less invasive bronchoscopic approaches have been designed to utilize these principles while avoiding the associated perioperative risks. The Endobronchial Valve for Emphysema PalliatioN Trial (VENT) posits that occlusion of a single pulmonary lobe through bronchoscopically placed Zephyr^® ^endobronchial valves will effect significant improvements in lung function and exercise tolerance with an acceptable risk profile in advanced emphysema.

**Methods:**

The trial design posted on Clinical trials.gov, on August 10, 2005 proposed an enrollment of 270 subjects. Inclusion criteria included: diagnosis of emphysema with forced expiratory volume in one second (FEV_1_) < 45% of predicted, hyperinflation (total lung capacity measured by body plethysmography > 100%; residual volume > 150% predicted), and heterogeneous emphysema defined using a quantitative chest computed tomography algorithm. Following standardized pulmonary rehabilitation, patients were randomized 2:1 to receive unilateral lobar placement of endobronchial valves plus optimal medical management or optimal medical management alone. The co-primary endpoint was the mean percent change in FEV_1 _and six minute walk distance at 180 days. Secondary end-points included mean percent change in St. George's Respiratory Questionnaire score and the mean absolute changes in the maximal work load measured by cycle ergometry, dyspnea (mMRC) score, and total oxygen use per day. Per patient response rates in clinically significant improvement/maintenance of FEV_1 _and six minute walk distance and technical success rates of valve placement were recorded. Apriori response predictors based on quantitative CT and lung physiology were defined.

**Conclusion:**

If endobronchial valves improve FEV_1 _and health status with an acceptable safety profile in advanced emphysema, they would offer a novel intervention for this progressive and debilitating disease.

**Trial Registration:**

ClinicalTrials.gov: NCT00129584

## Background

In this paper we describe the design of the Endobronchial Valve for Emphysema Palliation Trial (VENT). The primary objective of this study is to evaluate patients with heterogeneous emphysema treated with optimal medical management including pulmonary rehabilitation with or without implantation of the Emphasys (Emphasys Medical, Inc., Redwood City, CA) Endobronchial Valve (EBV).

Emphysema affects approximately 1.8% of the global population[1]. The disease is characterized by the gradual, irreversible breakdown of tissue and loss of elastic recoil within the lungs, leading to a reduction in expiratory airflow. As this disease progresses, the diseased, hyper-inflated regions of the lung continue to expand, imposing on the effective volume for more viable lung tissue to expand. As a result of these abnormalities in lung mechanics producing static and dynamic hyperinflation, patients exhibit a progressive increase in dyspnea, and reductions in exercise tolerance and quality of life.

Standard medical treatments for emphysema, which include smoking cessation, bronchodilators, pulmonary rehabilitation programs, and long-term home oxygen therapy, are aimed at providing improved exercise capacity and quality of life.

Surgical treatments for emphysema include single or double lung transplantation, lung volume reduction surgery (LVRS), and bulla resection. Transplantation is greatly limited by the small number of available donor organs and may not prolong survival. Lung volume reduction surgery, where the most diseased and hyper-inflated lung tissue is surgically resected, has been shown to offer relief to some subsets of patients with advanced emphysema when other treatment options have failed [2-6]. The National Emphysema Treatment Trial (NETT)[7] randomized 1,218 patients with advanced emphysema to either bilateral LVRS or standard medical therapy. In NETT, patients with bilateral, predominantly upper-lobe disease demonstrated sustained improvements in lung function, exercise tolerance and quality of life as compared to medical therapy alone. Moreover in the subgroup with upper lobe disease and low exercise performance post rehabilitation, a greater than 50% reduction in two year mortality following LVRS was demonstrated. Bulla resection is reserved for symptomatic emphysema patients presenting with a giant bulla which occupies more than 50% of the volume of a hemithorax.

The paradoxical effect of improving lung function by removing substantial amounts of diseased lung tissue suggests that breathlessness due to emphysema is a function of mechanical inefficiencies imposed by marked elevations in end-expiratory lung volume. Researchers have speculated that less invasive bronchoscopic approaches can utilize these principles of isolating and deflating the diseased, hyper-inflated regions to improve lung function and symptoms while avoiding the risks associated with LVRS.

### Rationale for VENT

**Figure 1:**
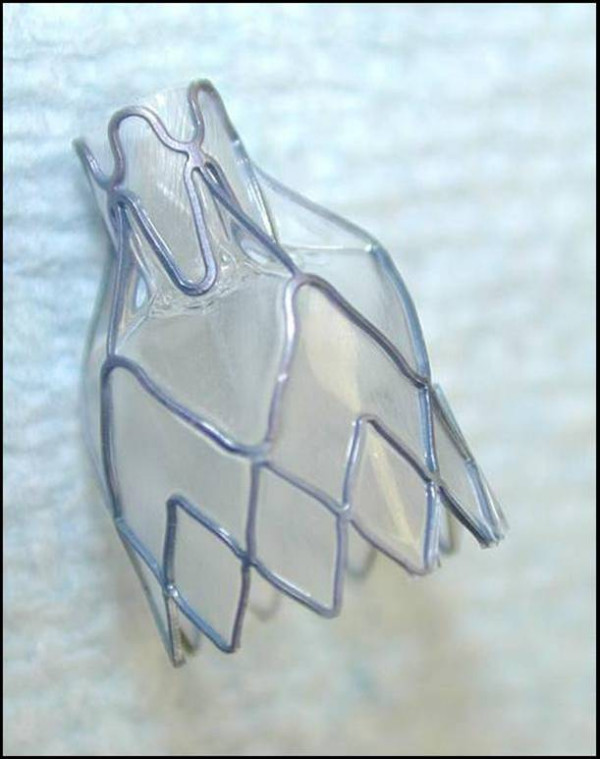
Zephyr^® ^Endobronchial Valve side view.

**Figure 2:**
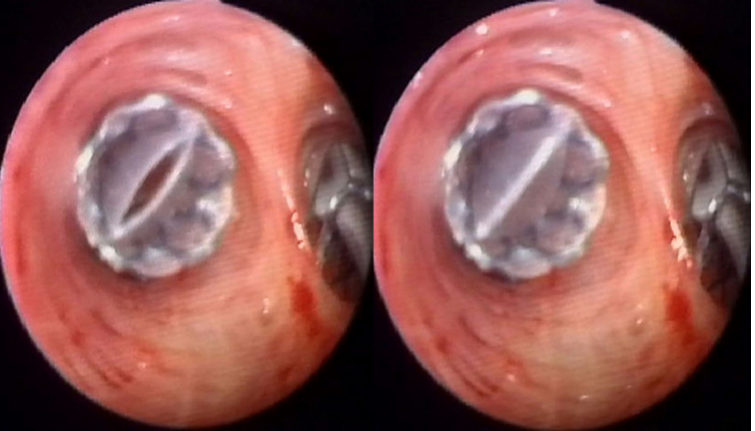
**Implanted Zephyr^® ^Endobronchial Valve end view**. The Zephyr^® ^Endobronchial Valve vents during expiration (left) and seals during inspiration (right) immediately after placement.

The Zephyr^® ^Endobronchial Valve (EBV) (Emphasys Medical, Inc., Redwood City, CA) is a bronchial implant incorporating a one-way valve that blocks the bronchial lumina leading to a targeted region of emphysematous lung (see Figure 1). The valve is supported by a stent-like self-expanding retainer that secures the EBV in place. In order to provide a complete seal during inspiration, the retainer is encased in a silicone membrane. The one-way valve allows gas to vent from the isolated lung segment during exhalation while preventing air from refilling the isolated lung area during inhalation (see Figure 2). The one-way valve is also designed to allow mucus to be expelled in order to reduce the likelihood of post-obstructive infection and to be removable if clinically indicated.

Non-controlled pilot studies using similar or earlier generation devices have been reported, showing improvement in lung function in short term follow-up evaluations (1–3 months after EBV implantation)[8]. Reported complication rates were favorable with respect to LVRS although pneumothorax incidence was 6.1% at 90 days.

VENT is the first prospective, randomized, multi-center trial to study endobronchial valves. The primary objective of VENT is to assess the safety and efficacy of EBV implantation combined with pulmonary rehabilitation, compared to optimal medical management with pulmonary rehabilitation, in patients with heterogeneous emphysema[9]. Additionally, the study is intended to identify patient characteristics or other procedural covariates that may affect the outcome of EBV placement and to shed further light on EBV mechanisms of improvement.

## Methods

### Overview

VENT is a two-arm, randomized, controlled, multi-center trial designed to study the safety and efficacy of the EBV implantation procedure, and the ability of the EBV to produce sustained improvement of symptoms in emphysema. The study sample size was a minimum of 270 subjects with heterogeneous emphysema, with randomization at a ratio of 2 to 1 (*i.e., *two patients were randomized to the study device treatment arm for every one randomized to the control group).

Given the similarity in intended treatment effect between LVRS and EBV implantation, combined with a goal of allowing easy comparisons between the results of the NETT and VENT, the VENT design largely followed that of the NETT. The inclusion/exclusion criteria were similar and both studies required pulmonary rehabilitation prior to randomization in order to maximize function prior to intervention. Both studies allowed treatment of the upper or lower lobes based on CT analysis; however, the NETT required bilateral (left and right lung) treatment whereas VENT treatments were unilateral only. Patient randomization in VENT was stratified per the subgroups identified in the NETT according to lung treatment region (upper versus lower) and post rehabilitation pre-treatment exercise capacity (low versus high).

In addition, the VENT study design conformed to the consensus recommendations of an independent panel of physician specialists convened by the U.S. Food and Drug Administration in February of 2003 to provide study design input for emerging bronchoscopic treatments for emphysema[10]. Questions considered at the panel included appropriate control groups, safety assessment, appropriate outcome metrics, and length of follow up.

A double-blinded sham controlled study design was considered for VENT, but was determined to be unsuitable for a number of reasons. Since the implants are radiopaque, it would be difficult to maintain the blind. Post-procedure chest x-rays are mandated by the protocol and are often clinically indicated along with CT scans during the management of the patient during the follow up period. For example, COPD exacerbations, a frequent occurrence in patients of this severity, are often seen with radiographs in the local emergency room. Maintaining the blind from emergency room physicians and radiologists would be problematic. Bronchoscopy during the follow up period would also un-mask the treatment arm. A patient may cough up a valve after implantation, thereby breaking the blind. Additionally, treatment decisions may differ based on the presence of an implant, forcing primary caregivers to unblind study subjects for evaluation and management of adverse events. Even if sham bronchoscopies were performed, patient recall of bronchoscopy procedures during moderate sedation is present in many cases. A sham procedure becomes less important as more objective effort-independent lung function parameters are included in the comparative analysis (e.g., residual volume measurements). Lastly, performing an unnecessary sham bronchoscopic implant in control patients with no chance of benefit, given their fragile health status, was considered to carry unacceptable risk.

A Clinical Events Committee (CEC) consisting of two pulmonologists and two thoracic surgeons adjudicates all reported complications. The CEC characterizes the severity of each reported event (mild, moderate, severe) and determines whether the event is related to the endobronchial valve or procedure (not related, remote possibility, possible, probable, unknown). Adjudicated complications are summarized and presented to an independent Data and Safety Monitoring Board (DSMB) consisting of two pulmonologists, two thoracic surgeons, and a biostatistician. The DSMB establishes pre-defined safety stopping rules and meets regularly to determine whether the study safety profile warrants continuation of the study.

### Primary outcome measures

The co-primary effectiveness endpoints are the mean percent change in both forced expiratory volume in one second (FEV_1_) and distance traveled in the six minute walk test (6 MWT) in the treatment group (EBV implantation) as compared to the control group (optimal medical management) at 180 days after randomization. The primary safety endpoint is a comparison of major complications between the two groups over the initial follow-up period of 180 days. Additionally, patients will be followed for up to 3 years post randomization for long term safety assessment.

### Secondary outcome measures

Disease specific quality of life (as measured by St. George's Respiratory Questionnaire), exercise capacity as measured by incremental cycle ergometry, daily supplemental oxygen requirement, and dyspnea (as measured by the modified Medical Research Council (mMRC) dyspnea scale, (see Table 1) are secondary efficacy endpoints to be assessed at baseline and 180 days post randomization. Appropriate adjustments will be made to account for impact on Type I error[11].

**Table 1:** Outcome measures

**Co-primary efficacy endpoints**	Mean % change in FEV_1 _and 6 MWT in the treatment group as compared to control group at 180 days post randomization.
**Primary safety endpoint**	Major Complications Composite at 180 days post randomization.
**Secondary efficacy endpoints**	Mean absolute change in: - St. George's Respiratory Questionnaire - Maximal work load as measured by cycle ergometry - Daily oxygen requirement - mMRC
**Secondary safety endpoints**	Complications (type, timing, and severity, including Kaplan-Meier survival analysis) Device-related adverse events during procedure hospitalization Device-related adverse events post discharge

Abbreviations: FEV_1 _= forced expiratory volume in one second, 6 MWT = six minute walk test, RV = residual volume, DLco = diffusion of the lung for carbon dioxide, BODE index = body-mass index, airflow obstruction, dyspnea, and exercise capacity index, EBV = endobronchial valve, mMRC = modified Medical Research Council dyspnea scale

### Patient recruitment

Study sites were encouraged to advertise for local recruitment, and web-links to study information were provided to relevant organizations (*e.g.: *the American Lung Association and the National Emphysema Foundation). Additionally, enrolling physicians were given written study information and a slide presentation for referring physicians in their area.

### Patient consent

Patients identified as potential participants were provided detailed explanations of the study and were asked for informed consent prior to initial screening. The patient consent form was approved by the Institutional Review Board (IRB) of all study sites, and failure to provide informed consent rendered the patient ineligible for the study.

### Screening assessments and high resolution computed tomography (HRCT)

Consented patients began an initial medical screening to assess preliminary eligibility including demographic data, medical history, physical exam, and inclusion/exclusion criteria screening. The procedures for assessment and determination of patient eligibility are outlined in Table 2 and detailed below. Further screening included spirometry, plethysmography, diffusing capacity, exercise tolerance, and a high resolution computed tomography (HRCT) scan of the chest.

**Table 2:** Screening and eligibility procedures

1.	Patient referred to study physician
2.	Patient consent obtained
3.	Screening Assessment 1: medical history, supplemental oxygen use and physical exam
4.	Determine if patient meets eligibility criteria
5.	Screening Assessment 2: electrocardiogram, spirometry, plethysmography, DLco, 6 MWT, Computed Tomography Scan, PaO_2_, PaCO_2_, arterial saturation, continine level, alpha-1 antitrypsin concentration
6.	Determine if patient continues to meet eligibility criteria
7.	Patient enters and completes pulmonary rehabilitation program
8.	Baseline measurements taken: medications, supplemental oxygen use, physical exam, electrocardiogram, spirometry, plethysmography, DLco, 6 MWT, cycle ergometry, quality of life surveys, chest x-ray, ventilation/perfusion (V/Q) scan, PaO_2_, PaCO_2_, arterial saturation, blood electrolytes, liver profile, renal profile, CBC, pregnancy test (where appropriate), and continine level
9.	Final determination of patient eligibility
10.	Randomization to Control or Treatment arm of the VENT study.

Abbreviations: DLco = diffusion of the lung for carbon monoxide, 6 MWT = six minute walk test, PaO_2 _= arterial pressure of oxygen, PaCO_2 _= arterial pressure of carbon dioxide, CBC = complete blood count.

The standardized volume acquisition CT was performed under the auspices of the imaging core at the David Geffen School of Medicine at UCLA. All images were acquired on multi-detector scanner platforms whose acquisition parameters were standardized to ensure similar image noise characteristics. Two sequences were acquired supine at suspended TLC and at RV, each in a single breathhold. The two sequences were reconstructed in both thick (5 or 10 mm) and thin (1.25 to 3 mm) series. Other sample acquisition parameters were customized for each site based on the scanner being utilized (e.g.: for the Siemens Sensation 16 scanner, the settings would be 120 kVp, 80 effective mAs, 0.5 sec. rotation time, 16 × 0.75 mm collimation, and 18 mm/rotation table feed with pitch 1.5).

The inclusion criteria identified patients with severe heterogeneous emphysema who have not previously undergone LVRS or EBV implantation and who were able to complete all the assessment and follow-up procedures. Candidates for this study were required to meet all of the inclusion criteria described in Table 3. The exclusion criteria were designed to identify potential study subjects that were unlikely to benefit from the treatment due to pre-existing conditions, or were unable to complete all of the follow-up assessments. Candidates were excluded from the study if any of the conditions listed in Table 4 were present.

**Table 3:** VENT inclusion criteria

**History and physical**	Age from 40 to 75 years. BMI ≤ 31.1 kg/m^2 ^(men) or ≤ 32.3 kg/m^2 ^(women). Stable with < 20 mg prednisone (or equivalent) daily. The patient has no child bearing potential OR a negative pregnancy test in a woman of childbearing potential.
**HRCT scan**	Patient diagnosed by HRCT Core Lab with eligible heterogeneous emphysema.
**Pulmonary function**	FEV_1 _< 45% of predicted value. TLC > 100% predicted. RV > 150% predicted.
**Blood gas**	PaCO_2 _< 50 mm Hg (Denver < 55 mm Hg). PaO_2 _> 45 mm Hg (Denver > 30 mm Hg) on room air.
**Exercise**	Post rehabilitation 6-minute walk of ≥ 140 meters.
**Smoking**	Nonsmoking for 4 months prior to initial interview and throughout screening. Plasma continine level < 13.7 ng/ml (or arterial carboxyhemoglobin < 2.5% if using nicotine products).
**Consent**	Patient has provided written informed consent using a form that has been reviewed and approved by the IRB/EC.
**Rehabilitation and follow-up**	The patient is willing and able to complete protocol required baseline assessments and procedures. The patient agrees to all protocol required follow-up intervals.

Abbreviations: BMI = body-mass index, HRCT = high resolution chest computed tomography, FEV_1 _= forced expiratory volume in one second, TLC = total lung capacity, RV = residual volume, PaO_2 _= arterial pressure of oxygen, PaCO_2 _= arterial pressure of carbon dioxide, IRB = institutional review board, EC = Ethics Committee.

**Table 4:** VENT exclusion criteria

**HRCT scan**	An HRCT Emphysema Score of 4-4-4 in the right lung or 4-4 in the left lung. Evidence of large bullae (encompassing >30% of either lung) in a non-target lobe.
**Pulmonary function**	FEV_1 _< 15% predicted value. DLco < 20% predicted value. Clinically significant bronchiectasis. Pulmonary nodule requiring surgery. History of recurrent respiratory infections (>1 hospitalization in the last year). Clinically significant (> 4 tablespoons per day) sputum production.
**Cardiovascular and exercise**	Dysrhythmia that might pose a risk during exercise or training. Congestive heart failure within 6 mo and LVEF < 45%. Resting bradycardia (< 50 beats/min), frequent multifocal PVCs, complex ventricular arrhythmia, or sustained SVT. Myocardial infarction within 6 mo and LVEF < 45%. Clinical suspicion or proven history of pulmonary hypertension. History of exercise-related syncope. Patient is unable to complete 3 minutes of unloaded peddling on cycle ergometer.
**Surgical**	Prior lung transplant, lung volume reduction surgery, median sternotomy, bullectomy or lobectomy.
**General medical**	Unplanned weight loss of >10% usual weight in 90 days prior to enrollment or total body weight < 70% of ideal body weight. Alpha-1 antitrypsin deficiency. Fever, elevated white cell count, or other evidence of active infection. Evidence of systemic disease or neoplasia expected to compromise survival during 5-yr period. Any disease or condition that interferes with completion of initial or follow-up assessments. Patient is currently enrolled in another clinical trial or has been previously enrolled in the VENT Trial for which protocol required follow up is not complete.

Abbreviations: HRCT = high resolution chest computed tomography, FEV_1 _= forced expiratory volume in one second, DLco = diffusion of the lung for carbon monoxide, LVEF = left ventricular ejection fraction, PVC = premature ventricular contractions, SVT = supraventricular tachycardia.

In the NETT, all HRCT data was analyzed visually by study site radiologists, and the lung was analyzed by zones that represented 33% of each lung. Visual grading of lung destruction due to emphysema has been shown to be highly dependent on the radiologist performing the analysis, and to be much less consistent than quantitative computer analysis[12]. The NETT study has subsequently applied computer based analysis to the HRCT scan data, although using the same lung zone convention that was establish for the radiologist reads. For this reason, automated computer analysis of the thick section CT scan data taken prior to randomization was used in VENT to determine patient eligibility for the study as well as to determine implant targeting for those patients in the treatment arm. The core radiology laboratory established CT scan acquisition quality standards to ensure that all scans, regardless of study site location, were taken under repeatable and consistent conditions. In addition, the core lab subdivided the lung into lobes and provided emphysema scoring on a lobar basis. Unlike the NETT study which used a zonal scoring system that did not correspond to lobar anatomy, the lobar scoring in VENT allowed targeting of the EBV implantation procedure to the most emphysematous lobe.

**Figure 3:**
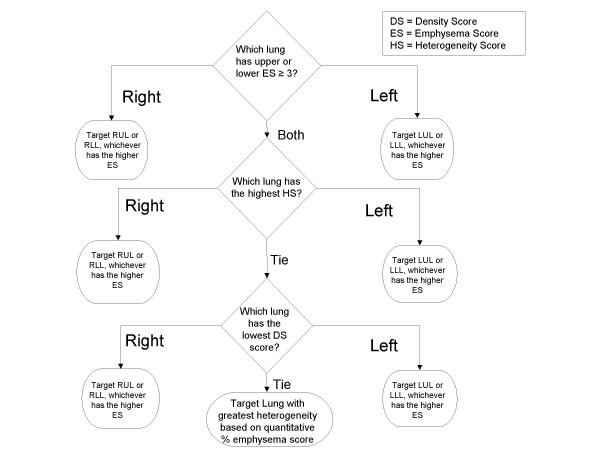
**Lobar treatment targeting algorithm**. A single lobe for treatment is selected by determining the highest emphysema score (ES). If emphysema scores are identical between lobes on both lungs, then the lung with the most heterogeneity is selected. The heterogeneity score (HS) is derived by subtracting the best lobe score from the worst lobe score in a single lung. If both lungs are equally affected with emphysema and heterogeneity, the computer generated absolute % density score (DS) is used to select the most affected lobe for targeting.

The baseline CT scan was provided to the CT core lab for analysis using a modification of the system used in the NETT study, a system adapted from prior work by Goddard et al[13], Bergin et al[14], and Bankier at al[12]. In the system used in VENT (see Table 5), the extent of emphysematous destruction was graded on a lobar level on a scale of 0 to 4 and called the emphysema score (ES). Because the intent of EBV treatment in the VENT trial was to completely isolate a targeted lobe, each lobe was graded individually according to the percentage area that demonstrated changes suggestive of emphysema, specifically, low attenuation, lung destruction, and vascular disruption. Using a predefined formula (see figure 3), the lobe targeted for isolation with EBV devices was determined by the core lab radiologists for each patient based on the CT scan analysis. The core lab radiologists were blinded to any clinical or physiological information on the patients.

**Table 5:** Lobar emphysema scoring

% of Parenchyma with Abnormalities Suggestive of Emphysema	Emphysema Score
0	0
1–25%	1
26–50%	2
51–75%	3
>75%	4

### VENT study treatments

#### Medical treatment and pulmonary rehabilitation

After preliminary determination of eligibility, all subjects received an optimal medical management program. Optimal medical therapy was defined, for the purposes of this protocol, as maximal medical treatment for stable chronic obstructive pulmonary disease (COPD) as presented in the 2001 National Institutes of Health/World Health Organization Global Initiative for Chronic Obstructive Lung Disease (GOLD) guidelines[15]. As recommended in the GOLD standards, each patient enrolled in the study received therapy consisting of the following components: education and smoking cessation support, pharmacological treatments including bronchodilators and influenza and pneumococcal vaccinations, non-pharmacological treatment including a 6–8 week pulmonary rehabilitation program and oxygen therapy if needed.

Pulmonary rehabilitation was an essential component of the pre-randomization optimal medical management program. Pulmonary rehabilitation has been shown to significantly improve exercise capacity and quality of life in COPD[16]. A pulmonary rehabilitation program was required for both control and treatment patients in the NETT study, and thus its inclusion in VENT allows more accurate comparisons between the results of both trials. Pulmonary rehabilitation prior to randomization was required in VENT to insure that any outcome differences between the treatment and control groups were due to the effect of the device rather than due to a training effect. It also minimized the chance for differences in health care exposure and treatment differences between randomly assigned treatment groups. Upon successful completion of the pulmonary rehabilitation program, a full baseline assessment was performed in each patient as outlined in table 2. If the patient continued to meet all protocol entry criteria shown in Tables 3 and 4, the patient was randomized to either the treatment or control group.

#### Control group

Once patients were randomized, the control group continued to receive optimal medical care at the participating center. Exercise continued at a minimum frequency of three times weekly. The control patients received the same follow-up as the treatment arm, including an office visit in lieu of EBV implantation. At the time of the control arm office visit, all patients were provided with a study diary to be used as a memory aid throughout the study duration.

#### Treatment group

The study group received the EBV implantation procedure within three weeks of randomization, and continued optimal medical management and exercise similar to the control group.

The goal of the implantation procedure was to completely isolate a single diseased lobe of the lungs by implanting EBV devices occluding all bronchial lumina leading to the targeted lobe. Bilateral treatment was not allowed in this study and acceptable target lobes did not include the right middle lobe. A single segmental airway not isolated by valves may allow ventilation to the lobe, thus reducing the potential benefit of the EBV devices. EBV devices were placed at the lobar, segmental, or sub segmental levels in this order of preference, depending on the anatomy of the patient noted at the time of the procedure.

The EBV implantation procedure was performed with the patient under general anesthesia and on a ventilator, or under moderate sedation with unassisted breathing. All patients were given antibiotics before and for 7 days following the procedure. Anesthesia was administered according to standard local protocols for bronchoscopy. For general anesthetic procedures, a rigid bronchoscope in conjunction with a flexible bronchoscope or a flexible bronchoscope alone through an endotracheal tube was allowed for valve placement.

Patients in both control and treatment arms were monitored for 1 year, with scheduled assessments at 1, 2–3, 7–10, 30, 90, 180, and 365 days post-procedure. Follow-up assessment includes the same variables as in the baseline assessment (see Table 2) with the primary outcome measures defined from the 180 ± 14 day follow-up visit.

### Analysis

The power analysis is based on a pilot study in 38 patients in which FEV_1 _improved 14.9% ± 33.7 (mean ± SD) and 6 MWT distance improved 20.4% ± 41.5%. The samples size required to detect a 15% improvement in the treatment arm in FEV_1 _and a 17% improvement in 6 MWT distance at an alpha of 0.05 and a power of 0.90 is a minimum of 270 subjects with a 2:1 randomization to EBV procedure. Both of these values are above the threshold for minimal clinically important differences for these tests[17-19].

The study is designed as a superiority trial. For the two primary outcomes, the study is only considered a success if both endpoints show an improvement (one-sided test at p < 0.025). A one-sided test is appropriate to test for superiority. The actual probability of a false positive is lower than 0.025 given the imperfect correlation of the two endpoints. If change in FEV1 and change in 6 MWT were independent, the probability of a false positive would be 0.025*0.025 = 0.000625. In the pilot study generating the null hypothesis, the correlation coefficient between the change in FEV1 and 6 MWT was 0.3889. Given this r, the alpha for achieving a positive outcome is actually 0.005 (0.025*0.025^(1/(1+0.3889))). This is an assurance of avoiding a false positive well beyond convention.

The 2:1 randomization is chosen to enhance enrollment and increase the EBV population numbers in order to study covariates of treatment response. Although the 2:1 randomization increases the number of subjects required for the study, more robust subgroup comparisons may be possible.

For safety the 95% upper confidence interval for the Major Complication Composite rate delta between the EBV arm and the control arm is ≤ 30%.

The primary analysis cohort is intent to treat. As such, all 180 day missing data will be imputed using either a regression methodology if 30 and 90 day data are present or hot-deck imputation if there are not multiple datapoints available for a particular subject for regression analysis. Three unbiased imputations will be performed and the imputation resulting in the highest p-values will be selected as the final intent to treat dataset.

The initial analysis will evaluate the mean percent change between EBV and control in FEV_1 _and 6 MWT using an unpaired t-test if data are normally distributed or Mann-Whitney-Wilcoxon test if data require non-parametric testing. To determine if covariates impact the primary outcome variables, and to adjust for potential imbalances in randomization, both primary endpoints will be further tested via a multivariate model (SAS PROC MIXED). In order to reduce the possibility of over-specification of the model, the potential covariates listed in Tables 6, 7, 8, 9 will be subjected to a univariate screening procedure similar to that described by Hosmer and Lemeshow[20]. Derivations for these variables are provided in Table 10. Main effects and interactions with a p-value of 0.15 or less are allowed to enter a multivariate analysis using a mixed linear model (SAS PROC MIXED). The treatment effect must remain in the model in order to substantiate the one-tailed test of superiority. Those variables found to have statistically significant interactions with the treatment arm via the mixed model analysis will be further analyzed in follow-on sub-group analyses. In order to limit the impact of multiple hypothesis testing, univariate sub-set analysis will be performed using only those independent variables that remain in the mixed model as interactions with the treatment arm. For those analyses performed on the remaining independent variables, appropriate adjustments will be made to account for impact on Type I error[11]. Secondary endpoint analyses will follow the same methodology as described above.

**Table 6:** Plethysmography, spirometry and HRCT derived baseline potential covariates*

Variable	Plethysmography/Spirometry	HRCT at TLC	HRCT at RV	TLC and RV HRCT ^†^
RV	X			
RV % Predicted	X			
TLC	X			
TLC % Predicted	X			
RV/TLC	X			
VC	X			
FVC	X			
FVC % Predicted	X			
FEV_1_	X			
FEV_1 _% Predicted	X			
FEV_1_/FVC	X			
Destruction Score of Target Lobe		X	X	
Ipsilateral Heterogeneity^††^		X	X	
Whole Lung Heterogeneity^††^		X	X	
For RUL Treatment Subset only: RUL-RML Heterogeneity^††^		X	X	
Target Lobe Volume % of TLC		X		
Target Lobe Volume % of RV			X	
Max Destruction Score other than target		X	X	
Min Destruction Score other than target		X	X	
Target Lobe Destruction Score % TLC-RV Delta				X
Target Lobe Volume % TLC-RV Delta				X
Fissure Score^†††^		X		

*Variables marked in multiple columns will be calculated twice, once using the expiratory (RV) CT and once using the inspiratory (TLC) CT. All tests are at Baseline.

^†^Some variables require information from both the TLC and RV CT

^†† ^Heterogeneity scores are derived by subtracting the minimum destruction score from the maximum destruction score.

^††† ^Fissure Score is an integer scale from one to three base on visual scoring by core center radiologists (1 = Fissure Absent, 2 = Incomplete Fissure, 3 = Complete Fissure).

**Table 7:** Other baseline and procedure potential covariates

	Baseline	Procedure
Gender	X	
Age	X	
Site	X	
6 MWT	X	
Cycle Ergometry	X	
BMI	X	
DLCO % Predicted	X	
PaO_2_	X	
PaCO_2_		
Target Lobe		X
RUL Treatment		X
Upper vs. Lower		X
Right vs. Left		X
Valve Version*		X
Large Valve*		X
Small Valve*		X
Valve Combos*		X
Valve Expectorated*		X

*These potential covariates apply only to the treatment arm and do not have a control arm subset.

**Table 8:** Composite or other potential covariate interactions

Composite or Interactions	Definition
Baseline BODE	BMI, FEV_1_% predicted, MMRC, 6 MWT Composite Score
NETT Strata	Randomization Stratification by Baseline Cycle Ergometry and HRCT at TLC
NETT Strata by Right vs. Left	Randomization Stratification by Baseline Cycle Ergometry and HRCT at TLC, by Right or Left Lung
Lobar Exclusion by Fissure Interaction*	Fissure Score = 3 AND Lobar Exclusion = Yes vs. all others
Lobar Exclusion, Fissure, and RV % Predicted*	Lobar Exclusion = Yes AND Fissure Score = 3 AND RV % Predicted ≥ 200% vs. all others
Lobar Exclusion, Fissure, and RV % Predicted*	Lobar Exclusion = Yes AND Fissure Score = 3 AND RV % Predicted ≥ 200% vs. all others
Large Valve by Right vs. Left*	Right vs. Left Lung AND Large Only vs. Others
Small Valve by Right vs. Left*	Right vs. Left Lung AND Small Only vs. Others
Expectorated Valve Replaced*	Valve(s) Expectorated AND Lobar Exclusion = Yes vs. all others
Expectorated Valve Not Replaced*	Valve(s) Expectorated AND Lobar Exclusion = No vs. all others
Lobar Exclusion by Site*	Technical Success by Site
First Case by Site*	First Treatment Case by Site vs. All Others
Learning Curve by Site*	Experience Level: Case 1, vs. 2 & 3, vs. 4–6, vs. 7–9, vs. ≥10 by site.

*These potential covariates apply only to the treatment arm and do not have a control arm subset.

**Table 9:** 180 day follow-up CT potential covariate*

Variable	180 Day HRCT at TLC	Baseline and 180 Day HRCT at TLC	Baseline and 180 Day HRCT at RV
Target Lobe Atelectasis Score^†^		X	X
Lobar Exclusion^†† ^(yes/no)	X		

*These potential covariates apply only to the treatment arm and do not have a control arm subset.

^†^Atelectasis Score is a continuous variable from 0 to 100% and is defined as the % volume change in the target lobe from baseline to 180 day follow-up.

^††^Lobar Exclusion will be determined by the core lab radiologists and is defined as all airways into the target lobe are occluded by valves at day 180.

**Table 10:** Covariate variable derivations

**Variable**	**Definition**
RV	Residual Volume by Spirometry and Plethysmography (TLC - Max (FVC or VC))
TLC	Total Lung Capacity by Plethysmography
VC	Vital Capacity by Plethysmography
FVC	Forced Vital Capacity by Spirometry
FEV_1_	Forced Expiratory Volume in the First Second by Spirometry
Target Lobe	RUL, RLL, LUL, or LLL
RUL	RUL Target vs. Others
Density Score	Density Score by Lobe
Total Density Score	Sum of all Lobar Density Scores (including RML)
Ipsilateral DS Heterogeneity	Target - Max DS of Non-treated Lobes within Treated Lung [e.g.: RUL - Max(RML, RLL)]
Thorax DS Heterogeneity	Target DS - Max DS of All Non-treated Lobes [e.g.: RUL - MAX (RML, RLL, LUL, LLL)]
Target Lobe DS % Delta	(TLC HRCT - RV HRCT)/TLC HRCT Density Score of Target Lobe
Target Lobe Volume % Delta	(TLC HRCT Volume - RV HRCT)/TLC HRCT Volume of Target Lobe
Fissure Score	Categorical Fissure Assessment Surrounding Target Lobe
	1 = Absent
	2 = Incomplete
	3 = Complete
NETT Strata	1 = Upper-Lobe Predominance, Low Baseline Exercise Capacity*
	2 = Upper-Lobe Predominance, High Baseline Exercise Capacity
	3 = Non-Upper-Lobe Predominance, Low Baseline Exercise Capacity
	4 = Non-Upper-Lobe Predominance, High Baseline Exercise Capacity
Target Lobe Atelectasis Scores	% change in Target Lobe Volume at TLC and at RV between Baseline and 180-day HRCT
Lobar Exclusion	Yes, If all bronchial pathways to target lobe are sealed by valve(s) – adjudicated by HRCT Core Lab
Valve Combinations	1. Small ONLY
	2. Small AND Large
	3. Large Only
Large Valve	Any procedure with Only Large Valves vs. all others
Small Valve	Any procedure with Only Small Valves vs. all others

*Low Exercise Capacity by Cycle Ergometry ≤ 25 W for Women, ≤ 40 W for Men

Thirty, 90, and 365 day results will also be collected and reported. Post hoc analysis will use these datapoints to define the speed of treatment success if present.

## Discussion

Bronchoscopic lung volume reduction holds the promise of improving outcomes for carefully selected individuals with lung hyperinflation from heterogeneous emphysema. Pilot studies have been performed in small numbers of patients that demonstrate improvements in FEV_1 _and exercise tolerance [4,21-24].

The current trial serves dual purposes. The first was to treat enough of the hyper-inflated lung to achieve a statistically significant improvement in physiology and symptoms. While there is certainly limitation to the amount of lung that can be safely treated without suffering hypoventilation or respiratory failure, the optimal amount of lung that can be safely targeted remains unknown. An analysis of pilot studies has suggested that a single lobar treatment strategy may be a reasonable goal for obtaining meaningful physiologic improvements in some patients. However, the outcome of this study is not expected to achieve the same physiologic outcomes that might be obtained by LVRS that targets 20–30% of both lungs during a single operative procedure.

The second goal was to carefully evaluate the safety of the EBV procedure and post procedural course. Pilot studies have documented a high frequency of pneumothorax following the procedure. Whether this represents pleural disruption by valve placement, rupture of residual blebs subsequent to stress relaxation in non-treated segments or a delay in lung remodeling following significant atelectasis of targeted lung remains unknown. Some clues to the pathogenesis will be obtained by an observation strategy imbedded in the protocol. Treatment of pneumothorax with a thoracostomy tube will be reserved for pneumothoraces that enlarge or cause respiratory failure.

COPD outcome is heavily influenced by exacerbation frequency. Since EBV valves are foreign bodies in an airway that may have some baseline hyper-responsiveness, the frequency and severity of exacerbations and the frequency of post-obstructive pneumonia are important safety considerations that warrant a long follow-up time.

Advanced emphysema is characterized by destruction of lung parenchyma and augmentation of anatomic connections for air passage between adjacent lung lobules. Unfortunately for endobronchial lung volume reduction procedures, these collateral communications may traverse lobar fissures [25]. These peripheral communications between lobes then may allow for continued lobar ventilation despite completely occluded lobar airways. Because this collateral ventilation is not easily quantified by current detection methods, the outcome of EBV valve placement may be heterogeneous. Ultimately, secondary analyses will include responder analysis, in order to determine the proportion of patients with clinically meaningful responses.

Given the complex mechanical and gas exchange consequences of valve insertion, it is possible that the magnitude of volume reduction in a lobe may not be associated with changes in other meaningful physiologic or functional indices following the procedure. For example, the impact of valve placement on symptoms associated with dynamic hyperinflation or variations in gas exchange utilizing collateral channels may be associated with clinical improvements independent of changes in conventional resting pulmonary tests. The composite primary endpoint was chosen to include a functional exercise measure to capture such changes.

Ultimately, the future of the EBV valve will be determined by the balance between efficacy and safety. Since the valves are removable for mild or moderate adverse events, valve safety will be impacted by the frequency of severe adverse events that might affect survival in a compromised patient population. In fact, individuals who demonstrate improvements in lung function may also demonstrate an increase in exacerbation frequency. For this reason, quality of life indices including the Quality of Well Being Scale and the Saint George's Respiratory Questionnaire are considered integrative parameters which can determine the balanced impact of the intervention [26,27]. One strategy to minimize overall adverse response would be to remove valves earlier in the patients who do not have clinically important responses. If a meaningful outcome is measurable in the first few days after the procedure, future alternative targeting strategies might be employed to determine response at earlier times after randomization.

### Study limitations

One limitation of this study is the difficulty in performing a double-blind sham procedure study for reasons discussed earlier. As such, a potential placebo effect could influence the quality of life and exercise measurements. From the investigator's perspective the frequency of adverse events attributable to the valve will obviously be different for the control and valve cohorts. Common but potentially serious adverse events such as exacerbation frequency may be interpreted differently given the knowledge of treatment assignment. Despite attempts to standardize insertion protocol, there remains considerable variability related to delivery of general or moderate sedation anesthesia that could affect procedure time and possibly outcome. Finally, the rigid inclusion criteria and targeting algorithms utilized in this trial limit the discovery of other potentially effective emphysema treatment strategies. Given the many subtypes of emphysema, the outcome of this study may not generalize to all individuals with the disease.

The VENT study evaluates the effectiveness of endobronchial valve placement to reduce lung volume in emphysematous patients as compared to optimal medical management. Due to the similarities between this study and the NETT study of lung volume reduction surgery, efficacy and safety comparisons to those achievable with LVRS are anticipated.

## Competing interests

All investigators have received research support from Emphasys medical. CS is a consultant to Arriva Pharmaceuticals, Talecris, and ZLB Behring; is on the speaker's bureau of Baxter, Boehringer-Ingelheim, Glaxo-Smith Kline, Pfizer, Talecris and ZLB Behring; and has research grants with the NIH and the Alpha-1 Foundation on the subject of emphysema. FJFH is on the speakers' bureau of Altana, Astra-Zeneca, Boehringer-Ingelheim, Glaxo-Smith Kline, Olympus, Superdimension and ZLB Behring. GM is part owner of VIDA Diagnostics, a software company that performs image analysis mostly in CT scans. KLK has been a recent investigator for Altana, Pfizer, Boehringer-Ingelheim and a consultant for Olympus. JG has NIH grants on Computer aided diagnosis of lung disease in CT and is a co-director of MedQIA a software and imaging core services company. MN has received speaker's fees, advisory and consultancy honoraria from Astra-Zeneca, Glaxo-Smith Kline, Boehringer Ingelheim, and Merck. AE reports no competing interests with the subject of this paper. GJC has research grant funding from Boehringer-Ingelheim, Glaxo-Smith Kline, Shering-Plough, Aeris Therapeutics, Altana, and Roche and is a member of the Advisory Boards of Shering-Plough, Ostuka and Ortho-Biotech. FS has received less than $10,000 in consulting fees from Emphasys medical prior to participation in the clinical trial related to regulatory and protocol design issues. He has received consulting fees from Boehringer-Ingelheim, Glaxo-Smith Kline, Pfizer, Novartis, Astra-Zeneca and Sepracor and was a principle investigator on the NIH supported NETT.

## Authors' contributions

FCS participated in the design and coordination of the study. CS, FH, KLK, GM, AE, JG, MN, GJC and FCS participated in trial coordination and helped to draft the manuscript. All authors read and approved the final manuscript.

## Pre-publication history

The pre-publication history for this paper can be accessed here:



## Supplementary Material

Additional File 1
