# Supplementary material for: Design of the Endobronchial Valve for Emphysema Palliation Trial (VENT): a non-surgical method of lung volume reduction
Source: BMC Pulm Med. 2007 Jul 3;7:10. doi: 10.1186/1471-2466-7-10 (PMC1949836; doi:10.1186/1471-2466-7-10)
Supplement: Additional File 1 [file 1471-2466-7-10-S1.doc]

**APPENDIX**

The members of the VENT Study Research Group include: **Office of the Chair of the Steering Committee:** *University of Pittsburgh Medical Center, Pittsburgh:* F. Sciurba, R. Landreneau, M. Crespo, L. Lane, W. Slivka, C. Fuhrman, P. Ferson, C. Witt; **Clinical Centers**: *Allgemeines Krankenhaus Wien, Vienna:* W. Klepetko, A. End, P. Jaksch; *Asklepios Fachkliniken München-Gauting, Gauting*; F. Stanzel; N.Weber, K. Haeussinger; *Azienda Ospedaliera Umberto I, Ancona:* S. Gasparini, S. De Luca; *Baptist Memorial Hospital, Memphis:* E. Golden, M. Smith, G. Williams; *Beth Israel Deaconess Medical Center, Boston:* A. Ernst, D. Feller-Kopman, R. Garland, S. Ashiku, M. DeCamp; *Botsford Hospital Livonia:* G.Ferguson, T.Murphy Thompson; *Cedars-Sinai Medical Center, Los Angeles:* R. McKenna, Jr., C. Geaga, D. Kusuanco; *Centre Hospit. Univ* *de Grenoble, Grenoble:* Ch. Pison, B. Wuyam, G. Ferretti, I. Vivodtzev, C. Righini; *Centre Hospit. Univ. de* *Reims, Reims:* F. Lebargy, G. Deslee, S.Dury; *Charite' Campus Mitte, Berlin:* C. Witt, B. Schmidt; *Groupe Hospitalier Pitié Salpétrière, Paris:* J. Gonzalez-Bermejo, V. Trosini-Desert, T. Similowski, C. Beigelman, M. H. Becquemin; *Henry Ford Hospital, Detroit:* M. Simoff*,* R. Almario; *Hopital Beaujon, Paris:* H. Mal, A. Marceau, M. Fournier, O. Brugière, G. Dauriat; *Hôpital Calmette, Lille:* C. Marquette, S. Leroy, T. Perez, N. Bautin; *Hopital Civil, Strasbourg:* G. Massard, R. Kessler, A. Charloux, M. Y. Jeung, A. Jory; *Hôpital de Bois Guillaume, Rouen:* J. F. Muir, C. Viacroze ,D. Benhamou ,A. Cuvelier ,L. Molano; *Hôpital Larrey, Toulouse:* B. Degano, C. Hermant, S. Pontier, J. Rami, V. Chabbert; *Hôpital Nord, Saint Etienne:* J. M. Vergnon, C Veyret, F. Costes, L. De Matos; *Hopital Saint-Antoine, Paris :* M. Febvre, C. Chouaid, B. Lebeau; *Hôpital Sainte Marguerite, Marseille:* C. Pinet, H. Dutau, P. Thomas, M. Badier, J. Y. Gaubert; *Hospital Clínic de Barcelona, Barcelona:* A. Xaubet, A. Torres, C. Agustí, J. Barberà; *Hospital Universitari Son Dureta, Palma Mallorca:* A. Agustí, B. Togores, F. Renom, J.Sauleda, R. Ramos; *Inova Fairfax Institute of Research, Falls Church:* S. Nathan, S. Ahmad, L. Collazo, E. Battle; *Klinikum Bremen-Ost gGmbH, Bremen:* E.Hecker, J. Volmerig, T. Jahn; *Klinikum Nürnberg, Nürnberg:* J. Ficker, M. Wagner, T. Fink; *Mater Misericordiae University Hospital, Dublin:* J. Egan, J. Coyle; *Mayo Clinic, Rochester:* E. Edell, D. Midthun, J. Utz, B. Andrist, K. Meiras; *Medical University of South Carolina, Charleston:* C. Strange, G. Silvestri, E. Williams-Cummings; *Morton Plant, Clearwater:* E. Freilich, D. Amin, J. Masson, D. Orlando, L. Morgan; *National Jewish Medical & Research Center, Denver:*  R. Bowler, C. Wheeler, K. Mitchell; *New York Presbyterian, New York:* R. Maxfield, M. Ginsburg, P. Jellen, F. Brogan; *Otto-Wagner Spital, Vienna:* A. Valipour, R. Kohansal, C. Burghuber; *Palmetto Richland Memorial Hospital, Lexington:* D. Elton, A. Sy, E. McFarland, N. Haase, M. Stout; *Peoria Pulmonary Associates, Peoria:* W. Tillis, P. Whitten, J. Crabb, K. Hartwig, A. Scott; *Pulmonary Associates, Phoenix:*  B. Levine, J. Ross, D. Baratz, L. Fu; *Remington-Davis Clinical Research, Columbus:* E. Cordasco, J. Adamo, J. Botte; *Sarasota Memorial Hospital, Sarasota:* K. Voelker, T. Horiuchi, K. Hurwitz, G. Ferreira, K. Morgan; *Shands Hospital - University of Florida, Gainesville:*  M. Jantz, A. Deem, A. Fox; *Southern Arizona VA Health Care System, Tucson:* S. Campbell, T. Vincent, W. Shen; *Southern Illinois University, Springfield:*  S. Hazelrigg, T. Boley; *St. Francis Hospital, Tulsa:* W. M. Boomer, J. Goulet, C. Mullins; *St. Vincent’s Hospital Manhattan, New York:* J. Cicena, P. O’Neill; *Temple University Hospital , Philadelphia:* G. Criner, J. Travaline, W. Chatila, G. Jones; *Thoraxklinik-Heidelberg, Heidelberg:* F. Herth, R. Eberhardt; *Veritas Clinical Specialties, Topeka:* W. Leeds, G. Fair, L. Ludlow, S. Nicklin, C. Davis; *Tulane University Hospital, New Orleans:* K. Kovitz, S. Ditta; *UC Davis, Sacramento:* A. Chan, R. Allen, T. Albertson, B. Morrissey, M. Juarez; *University Hospital Antwerp, Edegem:* P. Germonpre, A. Janssens, W. De Backer; *University Hospital AZ-VUB, Brussels:* M. Noppen, J. De Mey, M. Meysman; *University of Iowa Medical Center, Iowa City:* G. McLennan, J. Ferguson, A. Ross, K. Sprenger, J. Keating; *University of Kentucky, Lexington:* M. Zgoda, R. Berger, J. McClung, R. Cortes; *University of Maryland, Baltimore:* M. Krasna, J. Britt, Z. Gamliel, I. Moskowitz; *University of Michigan Medical Center, Ann Arbor:* S. Gay, J. Konkle; *VA Medical Center, Houston:*  S. Goodnight-White, S. Soubra, P. Smithwick, K. Velamuri; **Other participants:** *Data Safety Monitoring Board:* C. Cooper, B. Badduke, E. Hsiao; *Clinical Events Committee:* R. Wise, B. Celli, A. Ciccone, R. Keenan, D. Gilman; *CT Core Lab: University of California, Los Angeles:*  J. Goldin, M. Brown, M. McNitte-Gray, I. da Costa, J. Ho; *Cost Effectiveness Core Lab: UCSD Health Outcomes Assessment Program*: R. Kaplan, T. Ganiats; E. Groessl, J. Harvey, B. Mulligan
